# Genomic prediction in plants: opportunities for ensemble machine learning based approaches

**DOI:** 10.12688/f1000research.122437.1

**Published:** 2022-07-18

**Authors:** Muhammad Farooq, Aalt D.J. van Dijk, Harm Nijveen, Shahid Mansoor, Dick de Ridder

**Affiliations:** 1 Bioinformatics group, Department of Plant Science, Wageningen University and Research, Wageningen, Gelderland, 6708PB, The Netherlands; 2 Molecular Virology and Gene Silencing Lab, Agricultural Biotechnology Division, National Institute for Biotechnology and Genetic Engineering (NIBGE), Faisalabad, Punjab, 38000, Pakistan

**Keywords:** Genomic Prediction, Machine Learning, Genomic Selection, Linear Mixed Models

## Abstract

**Background:** Many studies have demonstrated the utility of machine learning (ML) methods for genomic prediction (GP) of various plant traits, but a clear rationale for choosing ML over conventionally used, often simpler parametric methods, is still lacking. Predictive performance of GP models might depend on a plethora of factors including sample size, number of markers, population structure and genetic architecture.

**Methods: **Here, we investigate which problem and dataset characteristics are related to good performance of ML methods for genomic prediction. We compare the predictive performance of two frequently used ensemble ML methods (Random Forest and Extreme Gradient Boosting) with parametric methods including genomic best linear unbiased prediction (GBLUP), reproducing kernel Hilbert space regression (RKHS), BayesA and BayesB. To explore problem characteristics, we use simulated and real plant traits under different genetic complexity levels determined by the number of Quantitative Trait Loci (QTLs), heritability ( *h* ^2^ and *h* ^2^ *_e_*), population structure and linkage disequilibrium between causal nucleotides and other SNPs.

**Results: **Decision tree based ensemble ML methods are a better choice for nonlinear phenotypes and are comparable to Bayesian methods for linear phenotypes in the case of large effect Quantitative Trait Nucleotides (QTNs). Furthermore, we find that ML methods are susceptible to confounding due to population structure but less sensitive to low linkage disequilibrium than linear parametric methods.

**Conclusions: **Overall, this provides insights into the role of ML in GP as well as guidelines for practitioners.

## Abbreviations

ANN: artificial neural network

BLUPs: Best Linear Unbiased Predictions

GBLUP: Genomic Best Linear Unbiased Prediction

GP: Genomic Prediction

MLP: Multilayer Perceptron

QTL: Quantitative Trait Loci

QTN: Quantitative Trait Nucleotide

RF: Random Forest

RKHS: Reproducing Kernel Hilbert Spacing

SNP: single nucleotide polymorphism

SVM: Support Vector Machine

SVR: Support Vector Regression

XGBoost: Extreme Gradient Boosting

## Introduction

**Figure 1.**
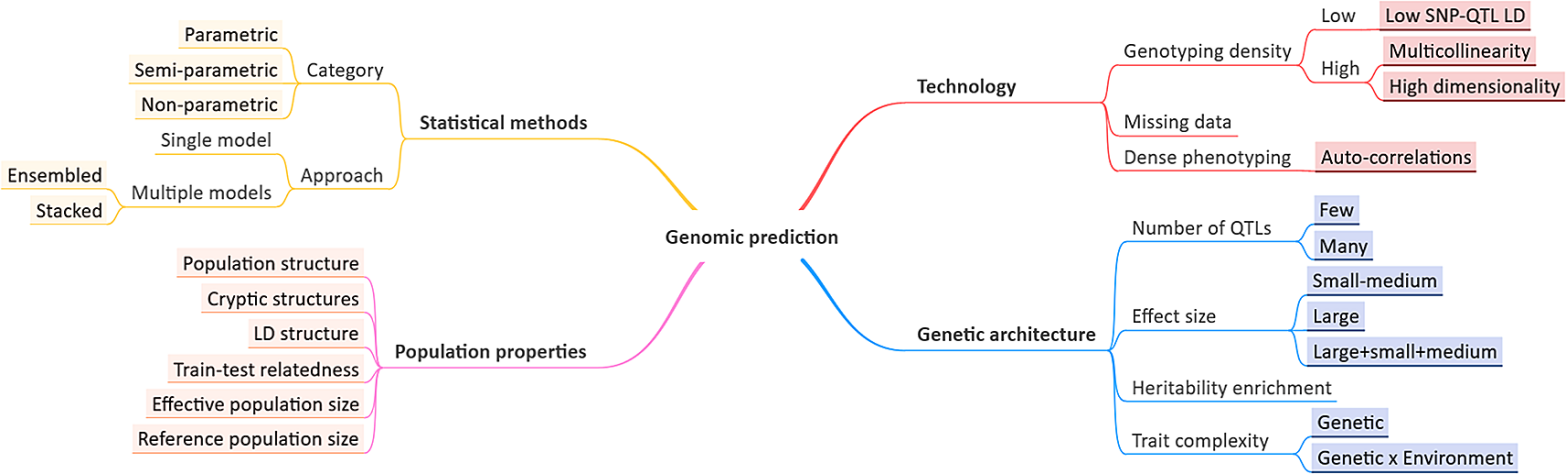
Genomic prediction characteristics. Factors affecting genomic prediction performance, often measured as correlation between true phenotype values and those predicted by a model.

The phenotype of an individual is based on its genetic makeup, the environment and the interplay between them. In plant and animal breeding, the genomic prediction (GP) model, using a genome-wide set of markers, is an integral component of the genomic selection-based approach. ^1^ A GP model is constructed on a reference population for which both genotypes and corresponding phenotypes are known, mostly employing a cross-validation strategy, and applied to related populations with only genotypes known. The total genomic value, estimated from the GP model, is used as a pseudo-phenotype to select the best parents for the next generation(s). In general, phenotypes differ from each other in terms of their genetic complexity, ranging from simple/monogenic to complex/polygenic. These differences impact the potential performance of GP. Complex traits are predominantly governed by a combination of additive and non-additive (e.g. dominant/recessive, epistatic etc.) allele effects, which makes GP challenging for these traits. ^2^ The genetic architecture of complex traits is characterized by moderate to large numbers of Quantitative Trait Loci (QTLs) with small to medium effect sizes and no or few large effect QTLs. ^3^ Moreover, the ratio of additive to non-additive genetic variance may differ even for closely related traits. Besides the actual genetic variance level, its distribution over the genome is also a determinant of the trait architecture. ^4^ Next to genetic architecture, population structure plays a role as well ( Figure 1): prediction accuracies are influenced by inconsistent relatedness among samples due to ancestral allele frequency imbalance among sub-populations (population structure) or cryptic structures, e.g. familial relationships; linkage disequilibrium (LD) structure, due to inbreeding or selection pressure; varying relatedness between training and test populations, e.g. over the course of a breeding cycle; and sizes of reference and effective populations. ^5^

Technological advances and statistical frameworks used bring new challenges ( Figure 1). Genotyping and/or phenotyping technologies can now generate millions of markers and thousands of phenotypic measurements, e.g. in time series, increasing the dimensionality of the prediction problem. For example, using a high-density SNP array (or imputing SNPs based on a low-density array) increases the likelihood of getting many markers in LD with the true QTL (high SNP-QTL LD). It can increase total explained variance, ^6^ but may induce multicollinearity among SNPs. Consequently, SNP selection prior to predictive modelling has been reported to provide superior performance compared to simply using a dense marker set. ^7^ In contrast, low-density genotyping can miss important SNPs in LD with, or weakly linked to, the QTLs, leading to inferior prediction performance. ^8^

Statistical genetics approaches have traditionally focused on formulating phenotype prediction as a parametric regression of one or more phenotypes on genomic markers, treating non-genetic effects as fixed or random in a linear equation. The resulting GP models are biologically interpretable but might yield poor performance for complex phenotypes, as linear regression fails to capture the more complex relations. ^9^ This approach also requires proper translation of prior knowledge on the genetics underlying phenotypes into parametric distributions. Although statistical distributions can help describe genetic architecture, devising a specific distribution for each phenotype is impractical. Therefore, many variations of linear regression were proposed by relaxing statistical assumptions; the main differences lie in their estimation framework and prior assumptions on the random effects (for an overview, see ‘Models’). Alternatively, machine learning (ML) offers a more general set of non-parametric methods that can model phenotypes as (non) linear combinations of genotypes. Moreover, these methods can jointly model the problem, e.g. strong learners can be stacked ^10^ or weak learners can be combined in an ensemble. Examples include Support Vector Machines (SVMs), (ensembles of) decision trees and artificial neural networks (ANNs). No statistical assumptions are required in advance; therefore, these methods should be able to pick up more complex genetic signals that are missed by linear models. The downside is the large amount of data required for learning these models from the data.

The performance of ML methods in GP problems has previously been compared using simulated and real phenotypes. Some were found to perform better under non-additive allelic activity ^11^^,^^12^; however, a clear link between simulated and real phenotypes is often missing, or only a specific breeding population structure is considered. For example, Barbosa *et al.* ^13^ compared the performance of ML and statistical methods in a simulated F _2_ population of 1,000 individuals and 2,010 SNPs using 26 simulated phenotypes. They varied the heritability and number of QTLs and included dominant and epistatic effects. They observed that ML methods performed better at low QTL numbers and hypothesized that a reason for this is that with fewer controlling genes, nonlinear epistatic interactions are more important. But it is still unclear if this is a general conclusion towards a population with different characteristics e.g. natural populations. Moreover, there are conflicting reports on performance of ML. ^11^^,^^14^ For example, ANNs have been reported to perform worse in some applications and are comparable to competing methods in others. ^12^^,^^15^ Ensemble decision tree methods, combining the output of a large number of simple predictors, have proven better for some traits but not for others. ^16^^–^^18^ Gradient boosting showed improved performances for many real traits ^19^^,^^20^ https://paperpile.com/c/ZyQHHy/b9hH+ha6M but was inferior to random forests on simulated datasets. ^18^ Furthermore, the impact of population structure and low SNP-QTL LD on the performance of ML methods is still unclear.

In this paper, we investigate which GP characteristics (genetic architecture, population properties and genotype/phenotype measurement technology) a priori point to a better performance for either traditional statistical approaches or ML-based methods. We compare GP performance of two ensemble methods, Random Forests (RF) and Extreme Gradient Boosting (XGBoost), to that of linear mixed models, GBLUP, BayesA, BayesB and RKHS regression with averaged multi-Gaussian kernels. We focus on typical applications in plant breeding to explore various GP characteristics, including the ratio of the total number of markers to the number of samples ( *p*/ *n*), genetic complexity, QTN effect sizes and distributions, linear (additive) vs. nonlinear (epistatic) heritabilities, sparse vs. dense genotyping and population structure.

## Methods

### Data

**Simulations**

In a first experiment, artificial genotypes were simulated, in combination with associated phenotype values. Genotype data was simulated for a diploid population with a minor allele frequency of 0.4, using a binomial distribution, where each allele was the outcome of a binomial trial. The genotype dataset was coded as {0=AA, 1=Aa, 2=aa}. To explore GP characteristics ( Figure 1), different levels of genetic complexity and dimensionality, defined as the ratio of total number of SNPs to the sample size ( *c* = *p*/ *n*), were simulated. For the high dimensionality scenarios, sample size was fixed at *n* = 500, because reference populations of this size are feasible for genotyping and phenotyping in genomic selection studies. Using values of *c* = {2, 10, 20, 40, 120}, the number of SNPs varied up to *p* = 60,000 (120*500). Similarly, for the low dimensionality scenarios, the number of SNPs was fixed at *p* = 500 and sample size was varied up to *n* = 3,000 to arrive at *c* = {1, 1/2, 1/4, 1/6}. Subsequently, Quantitative Trait Nucleotides (QTNs) were randomly selected from these simulated SNP sets to generate phenotypes. We selected either 5, 50, 100, *p*/2 or *p* QTNs, corresponding to a range of low to high genetic complexity, coupled with a narrow-sense heritability ranging from 0.1 to 0.7. A phenotype with a high number of QTNs and low heritability is more complex than one with few QTNs and higher heritability.

Phenotype datasets were generated using the simplePHENOTYPES v1.3.0 (RRID:SCR_022523) R package. ^21^ Linear polygenic phenotypes were simulated using additive modes of allele effects, as follows:

$$y=\beta_{1}{\mathrm{QTN}}_{1}+\beta_{2}{\mathrm{QTN}}_{2}+\beta_{3}{\mathrm{QTN}}_{3}+\ldots +\beta_{k}{\mathrm{QTN}}_{k}+\varepsilon$$ (1)

Here, *β* *_i_* describes the effect size of the *i ^th^* QTN, where **QTN** *_i_* is a vector containing the allele dosages for the *i ^th^* QTN for all samples. The residuals ( **ε**) were sampled from a normal distribution *N*(0, √(1- *h* ^2^)). The narrow-sense heritability ( *h* ^2^) determines the effect sizes: each QTN is assigned an effect size of *β* *_i_ = h* / *n,* referred to as ‘simulations with equal/uniform effects’ in the text. Smaller effect sizes can be generated by increasing the number of QTNs, thereby simulating higher genetic complexity. To further explore genetic complexity, we used equation (1) to generate another set of phenotypes where the first QTN is assigned a larger effect than others, 2 *h*, and the remainder still *h*/ ( *n-*1). Accordingly, another set of simulations was generated by sampling effect sizes from *N*(0, √ *h* ^2^).

For nonlinear phenotypes, broad-sense heritability was set at most to 0.8, so the distribution of residuals is *N*(0,√0.2). We considered only epistasis to induce nonlinearity, ignoring other factors such as dominance. Adding an additional term for epistasis to equation (1) results in:

$$y=\beta_{1}{\mathrm{QTN}}_{1}+\beta_{2}{\mathrm{QTN}}_{2}+\beta_{3}{\mathrm{QTN}}_{3}+\ldots +\beta_{k}{\mathrm{QTN}}_{k}+\beta_{e}\left({\mathrm{QTN}}_{e1}*{\mathrm{QTN}}_{e2}\right)+\varepsilon$$ (2)

The epistatic heritability ( *h* ^2^ *_e_*) was set analogous to the additive heritability ( *h* ^2^), such that *H* ^2^ = *h* ^2^ + *h* ^2^ *_e_.* The *additive* × *additive* epistasis model was used, with only a single pairwise interaction. The epistatic effect *β* *_e_* was sampled from *N*(0, √ *h* ^2^ *_e_*) and attributed to a single interacting pair of markers ( *e*1, *e*2) such that *β* *_e_* = *β* *_e1_* × *β* *_e2_.* We sampled this interacting pair from the set of additive QTNs; therefore, each interacting marker will always have some main effect. As for additive phenotypes, we also created nonlinear phenotypes with one large effect QTN. The total number of settings (scenarios considered in Table 1) for the simulated GP characteristics was 135 per phenotype class, i.e. linear and nonlinear. For each class, phenotypes were simulated with and without a large effect QTN. Thus, in total 810 (135 × 2 × 3) simulated phenotypic scenarios were generated, each having five independent phenotypic traits. ^22^^,^^23^ These will be referred to as ‘simdata’ in the text.

**Table 1.** Simulation scenarios permutations.

					Linear phenotypes ( *H* ^2^ = *h* ^2^)	Nonlinear phenotypes ( *H* ^2^ = *h* ^2^ + *h* ^2^ *_e_* = 0.8)
#Markers ( *p*) / #Samples ( *n*)	Ratio ( *c=p/n*)	#QTNs ( *q*)	#Markers ( *p*) / #QTNs ( *q*)	Ratio ( *d=p/q*)	*h* ^2^	*h* ^2^ + *h* ^2^ *_e_*
500/3k	0.17	5, 50, 100, 250, 500	500/5, 500/50, 500/100, 500/250, 500/500	100, 10, 5, 2, 1	0.1, 0.4, 0.7	0.7+0.1, 0.4+0.4, 0.1+0.7
500/2k	0.25	5, 50, 100, 250, 500	500/5, 500/50, 500/100, 500/250, 500/500	100, 10, 5, 2, 1	0.1, 0.4, 0.7	0.7+0.1, 0.4+0.4, 0.1+0.7
500/1k	0.50	5, 50, 100, 250, 500	500/5, 500/50, 500/100, 500/250, 500/500	100, 10, 5, 2, 1	0.1, 0.4, 0.7	0.7+0.1, 0.4+0.4, 0.1+0.7
500/500	1	5, 50, 100, 250, 500	500/5, 500/50, 500/100, 500/250, 500/500	100, 10, 5, 2, 1	0.1, 0.4, 0.7	0.7+0.1, 0.4+0.4, 0.1+0.7
1k/500	2	5, 50, 100, 500, 1k	1k/5, 1k/50, 1k/100, 1k/250, 1k/1k	200, 20, 10, 2, 1	0.1, 0.4, 0.7	0.7+0.1, 0.4+0.4, 0.1+0.7
5k/500	10	5, 50, 100, 2.5k, 5k	5k/5, 5k/50, 5k/100, 5k/250, 5k/5k	1k, 100, 50, 2, 1	0.1, 0.4, 0.7	0.7+0.1, 0.4+0.4, 0.1+0.7
10k/500	20	5, 50, 100, 5k, 10k	10k/5, 10k/50, 10k/100, 10k/250, 10k/10k	2k, 200, 100, 2, 1	0.1, 0.4, 0.7	0.7+0.1, 0.4+0.4, 0.1+0.7
20k/500	40	5, 50, 100, 10k, 20k	20k/5, 20k/50, 20k/100, 20k/250, 20k/20k	4k, 400, 200, 2, 1	0.1, 0.4, 0.7	0.7+0.1, 0.4+0.4, 0.1+0.7
60k/500	120	5, 50, 100, 30k, 60k	60k/5, 60k/50, 60k/100, 60k/250, 60k/60k	12k, 1.2k, 600, 2, 1	0.1, 0.4, 0.7	0.7+0.1, 0.4+0.4, 0.1+0.7

* Note: 1k=1000.

**Real datasets**

To compare trends observed in simulations with outcomes obtained with real traits, publicly available wheat genotype and phenotype data were taken from Norman, Taylor. ^24^ This includes 13 traits: biomass, glaucousness, grain protein, grain yield, greenness, growth habit, leaf loss, leaf width, Normalised Difference Vegetative Index (NDVI), physiological yellows, plant height, test weight (TW) and thousand kernel weight (TKW). This particular dataset was chosen as it contains a fairly large number of genotypes ( *n* = 10,375) each genotyped for *p* = 17,181 SNPs. The impact of population structure, training set size, marker density and its interaction with population structure was assessed in a study by the same authors ^25^ and GBLUP prediction accuracies were reported to saturate when training set size was greater than 8,000. We used the same settings, with five-fold cross-validation repeated for five times (training set size 8,300, validation set size 2,075).

The data was generated from a small-plot field experiment for pre-screening of germplasm containing some genotypes that are sown in multiple plots, thus containing spatial heterogeneity with correlation between closely located plots and imbalance in the number of phenotypes per genotype. Soil elevation and salinity, spatial coordinates and virtual blocks (made available on request by the authors) were taken as covariates:

$$y=\mathrm{Xb}+\mathrm{Zu}+Z_{g}g+\varepsilon$$ (3)

Here, **X** is the *n* × 4 design matrix for the fixed effects and overall mean, **b** is a 4 × 1 vector of fixed effects, i.e. soil salinity and elevation; **Z** is an *n* × 3 design matrix for non-genetic random effects **u**, i.e. range, row and block; **Z**_**g**_ is the *n* × *k* design matrix for genotypes **g** for a maximum of *k* replicates, and **ε** is an *n* × 1 vector of residuals. The Best Linear Unbiased Estimates (BLUEs) of genotypes were used for GP; in this way, we take care of the experimental design factors. Note that equation (3) does not contain any SNP information, instead only genotype accessions are used to obtain their adjusted phenotypes. ^22^^,^^23^

### Population structure analysis

To analyse the influence of population structure on the performance of different GP methods, we used a population of the *Arabidopsis thaliana* RegMap panel ^26^ with known structure, containing 1,307 accessions including regional samples (Extended Data, Figure S6 ^27^). Linear phenotypes were simulated using narrow-sense heritabilities *h* ^2^ = 0.1, 0.4 and 0.7, with equal effect QTNs. The genotypes, available from the *Arabidopsis* 250k SNP array, were further pruned for LD and minor allele frequency (MAF > 5%) using PLINK v1.9 (RRID:SCR_001757). ^28^ LD pruning was carried out using a window size of 500 markers, stride of 50 and pairwise *r* ^2^ threshold of 0.1, using the ‘--indep-pairwise’ command. This implies that a set of markers in the 500-marker window with squared pairwise correlation greater than 0.1 is greedily pruned from the window until no such pairs remain. This dataset will be referred to as ‘STRUCT-simdata’ in the text. ^22^^,^^23^

The effect of population structure was also assessed on real data: a genotype dataset of 300 out of the 1,307 RegMap accessions, phenotyped for the sodium accumulation trait with a strongly associated gene. ^29^ This should resemble one of our simulation scenarios, i.e. high heritability (e.g. *h* ^2^ = 0.7) with few QTNs (e.g. 5) of large effect. This dataset will be referred to as ‘STRUCT-realdata’ in the text. ^22^^,^^23^

To correct for population structure, we used principal components corresponding to the top ten highest eigenvalues as fixed effects in the models for GBLUP, RKHS regression, BayesA and BayesB. ^30^ Principal component analysis (PCA) was performed on the allele dosage matrix using the prcomp() method in R, with centering and scaling. For random forest and XGBoost, we used these top principal components as additional features in the models.

### Analysis of SNP-QTN linkage disequilibrium (LD)

To explore the impact of varying LD between SNP markers and actual QTNs on the performance of GP methods, we used two other datasets: one with real genotypes and simulated phenotypes, the other with real genotypes and real traits.

For the first dataset, we selected a natural population with minimal structure, balanced LD, genotyped at roughly equal genomic spacing and mostly inbred lines: the 360 accessions in the core set of the *Arabidopsis thaliana* HapMap population. ^29^ Genotype data of 344 out of the 360 core accessions was obtained from Farooq, van Dijk, ^31^ containing 207,981 SNPs. The phenotypes were simulated using one of the scenarios in the Section ‘Simulations’. The total number of SNPs was kept close to the number of samples and genetic complexity was kept low, to study the impact of SNP-QTN LD only. To this end, we simulated linear phenotypes with *h* ^2^ = 0.7 and 5 QTNs with equal effects. Linkage disequilibrium between SNPs was calculated as squared pairwise Pearson correlation coefficient ( *r* ^2^) using PLINK v1.9 (RRID:SCR_001757). ^28^ Input sets of 500 SNPs were selected randomly from pairs with either low LD ( *r* ^2^ ≤ 0.5) or high LD ( *r* ^2^ > 0.9); these two sets were used to train two prediction models using each GP method: one model was trained on the QTNs that were used to generate the phenotype, another on QTN-linked SNPs (closest on the genome) instead of the QTNs themselves, from the low or high LD SNPs pool. To avoid spurious correlations between SNPs in both models, non-QTN-linked SNPs were sampled from a different chromosome. We restricted the sampling of QTNs and the QTN-linked SNPs to chromosome 1, whereas the remaining non-QTN SNPs were sampled from chromosome 2. We refer to this dataset as ‘LD-simdata’ in the text. ^22^^,^^23^

For the second dataset, we used three soybean traits (HT: height, YLD: yield and R8: time to R8 developmental stage) phenotyped for the SoyNam population. ^32^ This dataset contains recombinant inbred lines (RILs) derived from 40 biparental populations and the set of markers have been extensively selected for the above traits. Moreover, high dimensionality is not an issue as the dataset contains 5,014 samples and 4,235 SNPs. We refer to this dataset as ‘LD-soy’ in the text. A complete list of datasets used in this study has been provided in Table 2 and achieved into public repositories. ^22^^,^^23^

**Table 2.** List of datasets. ^23^

ID	Description
simdata	Simulated dataset used to explore GP characteristics of trait genetic complexity, population properties and dimensionality. See Methods section 2.1.1 for details.
Wheat	Real wheat dataset from Norman, Taylor ^24^ containing 13 traits of varying genetic complexity. These traits are referred to by abbreviations: BM: Biomass, PH: Plant Height, NDVI: Normalised Difference Vegetative Index, LL: Leaf Loss, LW: Leaf Width, GY: Grain Yield, GL: Glaucousness, GP: Grain Protein, Y: Physiological Yellows, TW: Test Weight of grains, TKW: Thousand Kernel Weight, GH: Growth Habit, GR: Greenness
STRUCT-simdata	Real structured RegMap panel genotype data of 1,307 *Arabidopsis thaliana* accessions with simulated phenotypes data used to analyse the effect of population structure
STRUCT-realdata	A subset of the real *Arabidopsis thaliana* structured RegMap panel genotype data of 300 accessions with real phenotype data of the sodium accumulation trait used to analyse the effect of population structure
LD-simdata	An unstructured set of 360 accessions from the core set of the *Arabidopsis thaliana* HapMap population with known genotype data and simulated phenotype data to study the impact of LD
LD-soy	Real soybean dataset of with real phenotypes (R8, HT: height and YLD: yield) for studying the impact of low SNP-QTN LD ^32^

### Models

A wide range of statistical models have been proposed for GP. Most widely applied are Linear Mixed Models (LMMs), which use whole-genome regression to tackle multicollinearity and high-dimensionality with shrinkage during parameter estimation, employing either a frequentist approach, e.g. restricted maximum likelihood (REML), or Bayesian theory. ^33^ Below, we briefly describe the GP methods used in our experiments. For (semi) parametric methods, we used BGLR v1.1.0 (RRID:SCR_022522) with default settings of hyperparameters ^34^; for Random Forests, the ranger R package v0.14.1 (RRID:SCR_022521) ^35^; and for XGBoost, h2o4gpu v0.3.3 (RRID:SCR_022520). ^36^

**Parametric models**

***GBLUP***

The genomic best linear unbiased prediction (GBLUP) method uses a Gaussian prior with equal variance for all markers and a covariance matrix between individuals, called the genomic relationship matrix (GRM), calculated using identity by state (IBS) distances between markers for each pair of samples. ^37^ SNP effects are modelled as random effects that follow a normal distribution with zero mean and common variance, and are estimated by solving the mixed model equation:

y=μ+g+ε (4)

Here, **g** is an *n* × 1 vector of the total genomic value of an individual, captured by all genomic markers; **μ** is the overall population mean; and **ε** is an *n*-vector of residuals. The genomic values **g** and residuals were assumed to be independent and normally distributed as **g** ~ *N*(0, **G** *σ* ^2^ *_g_*), **ε** ~ *N*(0, **I** *σ* ^2^_ε_). Here **G** is the GRM, calculated using the rrBLUP v4.6.1 (RRID:SCR_022519) package ^38^ in R, providing variance-covariance structure for genotypes and **I** is the identity matrix. Due to the small number of estimable parameters, GBLUP is computationally fast but the assumption of normality only holds when most effects are close to zero and only a few are larger. The limitation of this approach is that it captures only linear relationships between individuals and assumption of equal variance for all marker effects may not be truly valid for many traits.

***Bayesian methods***

Several Bayesian methods with slight variations in their prior distributions have been proposed to model different genetic architectures ^39^ e.g. BayesA, using a scaled *t*-distribution; Bayesian LASSO or BL, ^40^ using a double-exponential; BayesCπ ^41^ and BayesBπ, ^1^ both utilising two-component mixture priors with point mass at zero and either a Gaussian or scaled *t*-distribution, respectively. To control the proportion of zero effect markers, the hyperparameter ‘π’ was set equal to 0.5, resulting in a weakly informative prior. For simplicity, we refer to BayesBπ as BayesB in the text. The model in equation (5) was solved for posterior means in both BayesA and BayesB with the only difference in priors of *β* *_j_*:

$$y=\mu +\sum_{j}^{J}x_{j}\beta_{j}+\varepsilon$$ (5)

Here, **μ** is the intercept, **x**_***j***_ is an *n-*vector of allele dosages for each SNP and *β* *_j_* is the effect of SNP *j* out of a total of *J* SNPs.

**Semi-parametric models**

Reproducing Kernel Hilbert Spaces (RKHS) regression is a general semiparametric method that models pairwise distances between samples by a Gaussian kernel and can therefore better capture nonlinear relationships than GBLUP. In fact, GBLUP is a special case of RKHS regression, with a linear kernel ^42^^,^^43^ https://paperpile.com/c/ZyQHHy/1oKK+NO1v. We used RKHS regression as a representative semi-parametric model, because it not only employs prior assumptions for random components in LMM equation (6), but also learns hyperparameters from the data itself:

$$y=\mu +\sum_{l=1}^{3}g_{l}+\varepsilon$$ (6)

In contrast to the GBLUP model ( 4), the RKHS regression model has three random genetic components $g=\sum_{l=1}^{3}g_{l}$ , such that **g** *_l_* ~ *N*(0, **K** *_l_σ* ^2^ *_gl_*); where **K** *_l_* is the kernel evaluated for the *l* ^th^ component. This kernel matrix **K** is used as genomic relationship matrix, where **K =** { *k* ( **x** *_i_* **, x** *_j_*)} is an *n* × *n* matrix of Gaussian kernels applied to the average squared-Euclidean distance between genotypes:

$$k\left(x_{i},x_{j}\right)=\exp\left(-b\left(\sum_{k=1}^{p}{\left(x_{\mathrm{ik}}-x_{\mathrm{jk}}\right)}^{2}\right)/p\right)$$ (7)

The kernel *k* **(x** *_i_*, **x** *_j_* **)** is a covariance function that maps genetic distances between pairs of individuals **x** *_i_* and **x** *_j_* onto a positive real value. The hyperparameter *b*, called the bandwidth, controls the rate at which this covariance function drops with increasing distance between pairs of genotypes. Tuning this parameter for range of values between 0 and 1 could be computationally inefficient. So, instead of tuning *b*, we used a kernel averaging method, ^42^ such that multiple kernels, corresponding to possible bandwidth values *b* = {0.2, 0.5, 0.8}, were averaged.

**Ensemble machine learning models**

***Random Forest***

The Random Forest (RF) regressor uses an ensemble of decision trees (DTs) that are each grown using bootstrapping (random sampling with replacement of samples), and a random subset of SNPs. The test sample prediction is made by averaging all unpruned DTs as;

$${\hat{f}}_{\mathrm{RF}}^{D}\left(x\right)=\frac{1}{D}\sum_{k=1}^{D}\tau \left(x,\psi_{k}\right)$$ (8)

Here ***x*** is the test sample genotype using an RF τ with *D* decision trees, for which $\psi_{k}$ is the *k ^th^* tree. An RF has a number of hyperparameters that need to be tuned, for which we used grid search using the caret v6.0.92 (RRID:SCR_021138) R package ^44^ https://paperpile.com/c/ZyQHHy/GaA1. We used 500 trees in the forest for all analyses and tuned ‘mtry’ and ‘nodesize’ hyperparameters to control tree shapes. The total number of SNPs randomly selected at each tree node, i.e. mtry, was selected from { *p*/3, *p*/4, *p*/5, *p*/6} and the minimum size of terminal nodes below which no split can be tried, i.e. nodesize, was selected from {0.01, 0.05, 0.1, 0.2, 0.3} times the number of training samples in each cross-validation fold.

***Extreme Gradient Boosting (XGBoost)***

We used XGBoost, a specific implementation of the Gradient Boosting (GB) method. Similar to the Random Forest, Gradient Boosting is an ensemble method, using weak learners such as DTs. The main difference is that an RF aggregates independent DTs trained on random subsets of data (bagging), whereas GB grows iteratively (boosting) by selecting samples in the subsequent DTs based on sample weights obtained in previous DTs, related to how well samples are predicted already by these previous DTs.

Hyperparameters were tuned using a grid search through five-fold cross-validation on each training data fold. We searched over max_depth = {2, 3, 4, 50, 100, 500}, colsample_bytree = {0.1, 0.2, 0.3, 0.5, 0.7, 0.9} and subsample = {0.7, 0.8, 0.9}.

**Performance evaluation**

Model performance was evaluated based on prediction accuracy, which was measured as the Pearson correlation coefficient ( *r*) between observed phenotypic values and predicted genomic values of the test population. For each model, five repeats of five-fold cross-validation were performed, so in total 25 values of *r* were used to compare performances. Statistical comparison between different models was performed by comparing prediction accuracies of each pair of models as a whole, i.e. on all values of *p/n* together using Wilcoxon rank-sum test.

### Assessment of trait nonlinearity

To link GP performance in simulation scenarios with performance on real data, an assessment of the nature of real traits (i.e. linear or nonlinear) was used. To obtain a proxy for linearity of the trait, we assumed that if a trait has a higher proportion of additive variance compared to other traits, estimated with the same model, it will be more linear. To verify this on our simulated dataset scenarios ( Table 1) for nonlinear phenotypes, we used the linear mixed model:

$$y=\mu +\left(g_{a}+g_{r}\right)+\varepsilon$$ (9)

Here **g** *_a_* defines a set of additive genotype effects such that **g** *_a_* ∼ *N*(0,σ ^2^ *_a_* **G**), where **G** is the genomic relationship matrix (GRM) calculated as described by VanRaden, ^37^ **g** *_r_* ∼ *N*(0,σ ^2^ *_r_* **I**) is the residual genetic effect and **ε** is a vector of residuals. The ratio of additive genetic variance to the residual genetic variance (σ ^2^ *_a_*/σ ^2^ *_r_*) was calculated for both the simulated dataset and real wheat traits. We tested our assumption on simulated phenotypes (Extended Data, Figure S1 ^27^), showing simulated amounts of non-additive heritability to indeed be negatively related to empirical additive heritability.

## Results

### ML outperforms traditional methods for GP

Previously, numerous GP methods were tested for different traits of varying genetic architectures using low or high density marker sets, but it is still unclear for which (class of) GP problems applying machine learning (ML) can be beneficial. ^9^ To investigate the role of underlying characteristics ( Figure 1), we generated an extensive set of simulated genotype-phenotype data (simdata: see Section ‘Simulations’). This data was analysed using the linear parametric methods GBLUP, BayesA and BayesB; the nonlinear semi-parametric regression method RKHS, using a Gaussian multi-kernel evaluated as average squared-Euclidean distance between genotypes ^42^; and popular nonlinear ML methods, i.e. support vector regressor (SVR), random forest regressor (RF), extreme gradient boosting (XGBoost) regression trees and a fully-connected feed forward artificial neural network i.e. Multilayer Perceptron (MLP). The simulations covered a variety of trait scenarios (from simple to more complex), as shown in Table 1. Simple oligogenic traits correspond to simulation scenarios with larger heritabilities, additive allele effects (linear) and small numbers of QTNs; complex traits can have both additive and non-additive allele effects (nonlinear) with small heritabilities and large numbers of QTNs. For linear phenotypes, narrow-sense heritability was set equal to broad-sense heritability and for the nonlinear phenotypes, the sum of narrow-sense and epistatic heritability was set equal to the broad-sense heritability. The extent of phenotypic linearity in both simulations and real datasets was calculated using the ratio of additive genetic variance to the residual genetic variance ( *σ* ^2^ *_a_*/ *σ* ^2^ *_r_*) from equation (9). In the results presented below, SVR and MLP were excluded because their performances were significantly lower than the tree-based ensemble ML methods (i.e. RF and XGBoost) on a subset of our simulation scenarios (Extended Data, Appendix I ^45^). Moreover, the applicability of neural networks/deep learning for GP in the feature space is still limited due to their high tendency toward overfitting under high-dimensionality until they are properly regularized or feature selection is employed. ^16^^,^^46^^,^^47^

**ML methods perform well for simple traits**

Many non-mendelian plant traits are fairly simple, where only one or a few QTLs explain a large proportion of phenotypic variance, called oligogenic traits. If these QTLs are identified by the GP model, prediction performance can be pretty high. In our simulations ( Table 1), this scenario is investigated using additive phenotypes with narrow-sense heritability ( *h* ^2^) equal to 0.7 and a total number of QTNs equal to 5. We then alternatively attribute equal effects to all QTNs, assign a larger effect to the first QTN in equation (1) compared to other the QTNs, or sample the QTN effects from a Gaussian distribution (see Section ‘Simulations’).

**Figure 2.**
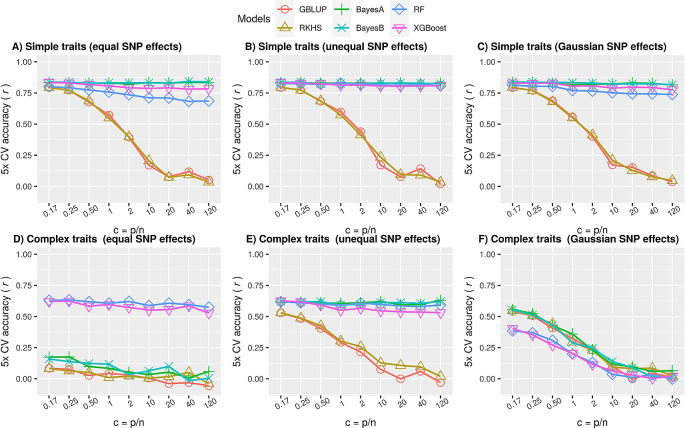
Comparison of prediction performances using simulated simple and complex phenotypes. Performance of parametric (GBLUP), semi-parametric regression (RKHS), parametric Bayesian (BayesA, BayesB) and nonparametric ML (RF and XGBoost) methods as average accuracy over *5*-fold cross-validation of test data. Here accuracy is defined as Pearson correlation coefficient between true and predicted values. Each panel is a subset of the simulated scenarios in ‘simdata’ for a particular heritability and #QTNs. The ratio of the number of markers to the number of samples ( *c* = *p*/ *n*) increases from left to right in each subplot. A) Simple traits, simulated as linear polygenic phenotypes with only additive effects such that #QTNs is equal to 5 and *h* ^2^ is 0.7, using equation (1), with all QTNs having equal effects. The largest standard error of mean for all values of *c* for each of the model was 0.023, 0.018, 0.007, 0.008, 0.018 and 0.009 for GBLUP, RKHS, BayesA, BayesB, RF and XGBoost respectively; B) similar to A, except one of the QTN had a large effect than others. The largest standard error of mean for all values of *c* for each of the model was 0.022, 0.022, 0.006, 0.007, 0.006 and 0.008 for GBLUP, RKHS, BayesA, BayesB, RF and XGBoost respectively; C) similar to A and B, except QTN effects were sampled from a Gaussian distribution; D) Complex traits, simulated as nonlinear polygenic phenotypes with both additive and epistatic effects such that #QTNs equal to *p*/2 and h ^2^ is equal to 0.4, using equation (2), such that all QTNs had equal additive effects. Two of the QTNs were attributed to the epistatic effect such that Broad-sense heritability was set to 0.8 ( *H* ^2^ = *h* ^2^ + *h* ^2^ *_e_* = 0.8). The largest standard error of mean for all values of *c* for each of the models was 0.03; E) similar to D, except one of the QTN had a large effect than others; F) similar to D and E, except QTN effects were sampled from a Gaussian distribution (see methods).

The results in Figure 2A, Figure 2B and Figure 2C illustrate that the performance of Bayesian methods and ML was significantly better (p value < 0.01; Extended Data, Table S1 ^48^) than that of genomic relationship-based methods (GBLUP, RKHS). The performance of ML methods was slightly poorer than that of Bayesian methods when all QTNs effects were equal ( Figure 2A) or sampled from a Gaussian distribution ( Figure 2C) but comparable when one of them had a larger effect size ( Figure 2B). Therefore, although not outperforming the other methods, ensemble ML methods seem to be reasonable choices for simple traits.

**ML methods outperform parametric methods for complex traits**

Complex polygenic traits may contain a large effect QTL along with many small to medium effect QTLs. ^49^ Despite assuming perfect LD between SNPs and their corresponding QTLs, their detection remains challenging through conventional univariate regression models that are followed by strict multiple testing corrections. Moreover, shrinkage of random effects towards zero in multivariate regression models restricts them from growing too large. Thus, many true small effects may be ignored in the analysis. SNPs may also have non-additive effects, which could cause a large amount of variance to remain unexplained and narrow-sense heritabilities to be low, when modelled by their linear action only.

This genetic complexity was simulated by increasing the number of QTNs, decreasing the narrow-sense heritability and keeping overall effect sizes equal, thereby letting the effect sizes per QTN become proportionally smaller. The QTNs were randomly chosen from the simulated SNPs pool by setting *k* equal to half of the total number of SNPs ( *p*/2) in equation (2), keeping equal effect sizes for all QTNs and *h* ^2^ equal to 0.4. Moreover, similar to simple traits, the other two scenarios, i.e. unequal effect sizes and normally distributed effect sizes, were also simulated. Two QTNs were randomly selected to have a fairly large pairwise interaction effect, corresponding to an epistatic heritability *h* ^2^ *_e_* equal to 0.4. The results in Figure 2D illustrate that ML methods significantly outperformed all methods for complex nonlinear phenotypes when all of the QTNs had equal effects (p-value < 0.01; Extended Data, Table S2 ^48^). Interestingly, when one of the QTN had a larger effect size or was attributed with most of the variance, the Bayesian methods performed on par with ML ( Figure 2E), but when the effect sizes followed a Gaussian distribution ( Figure 2F), ML was outperformed by the other methods. This confirms that parametric methods work well if the effects distribution matches the statistical prior assumptions. In reality, genetic variance may not be attributed to a single Gaussian for other than infinitesimal model, instead it could be decomposed into multiple distributions enriched in multiple chromosomal localisations defined by heritability models. ^50^ This phenotype complexity is usually unknown and difficult to accurately assess, which provides room for the ML methods.

**ML methods are generally suitable for nonlinear phenotypes**

For complex phenotypes, we observed that ML outperformed LMMs under highly polygenic phenotypes with epistatic effect and equivalent to Bayesian LMMs when at least one QTN had larger effect ( Figure 2D and E). To explore further, we investigated a range of additive and non-additive fractions of heritabilities, with or without a large effect QTN and from Gaussian distribution defined in our simulation scenarios ( Table 1).

For linear phenotypes with equal QTN effect sizes, performance of ML methods was poorer than that of Bayesian methods under all scenarios; with an increase in genetic complexity (lowering *h* ^2^ and increasing the number of QTNs), performance dropped below that of GBLUP and RKHS as well (Extended Data, Figure S2A ^27^). Therefore, ML methods are not beneficial for this setting. For nonlinear phenotypes however, ML outperformed all methods including the Bayesian methods for all scenarios (Extended Data, Figure S2B ^27^), with random forests generalizing the best. ML methods are thus best suited for nonlinear traits and do not necessarily need large main effects to be present. Note that although RKHS regression has been reported to better capture epistatic relationships between markers, ^43^ it did not perform well in our simulations; perhaps it needs more careful tuning of the bandwidth of the Gaussian distributions, rather than using multi-kernel averaging or require matching prior allele effects distributions (see Discussion, ‘Tree-based ensemble ML methods are a reasonable choice for GP’).

For the phenotypes explained by a large effect QTN and many small effect QTNs (Extended Data: Figures S3A and S3B ^27^), Bayesian methods perform comparable to ML methods for both linear and nonlinear phenotypes under all simulation scenarios, although RF gave slightly better performance for nonlinear phenotypes with large epistatic heritability (for *h ^2^_e_* = 0.7) and dimensionality ( *p/n* > 2). This could be because the large effect QTN explains most of the additive variance and is easily picked by Bayes and ML methods, but RF has the added advantage of picking up the nonlinear signal, when main effects got smaller with the increase in number of QTNs. XGBoost gave relatively poor performance, especially at smaller heritabilities (0.1 and 0.4) and larger *p*/ *n* ratios, while GBLUP and RKHS regression performance was consistently poor in all scenarios.

For both linear and nonlinear phenotypes (Extended Data: Figures S4a, and S4b ^27^), the ensemble ML methods were still superior over BLUPs and comparable to Bayes when effect sizes were sampled from a Gaussian distribution for a small number of QTNs (e.g. *q* = 5, h ^2^ = 0.7, h ^2^_e_ = 0.1), but the advantage diminishes when *q* increases and approaches the infinitesimal model i.e. *q = p.*

In conclusion, our simulation results indicate that ML works well when a fair proportion of broad-sense heritability is contributed by nonlinear allele effects or a few large effect QTNs.

**ML performance is robust to high-dimensional GP**

Genomic prediction is usually employed on a genome-wide set of markers to yield total genomic value, but the training population size is limited, i.e. a high dimensional problem. This results into more statistical power to detect QTLs with many SNPs in LD but comes with obscured genetic variance when added together. Consequently, it leads to an overestimation of allelic variances or genomic relationships, overfitting on training samples and reduced performance on unseen data. To investigate the susceptibility of different GP methods for this issue, we analysed how prediction accuracy varied depending on the ratio of markers vs samples (c = *p*/ *n* > 1).

In general, the results with different simulation settings of ‘simdata’ for linear phenotypes show that performance is negatively related to an increase in dimensionality when main effects got smaller due to decreasing heritability or increasing total number of QTNs (Extended Data: Figure S2A, Figure S3A and Figure S4A ^27^). This implies that for simple traits having one or few large effect QTNs ( Figure 2A to C), performance degradation is not a severe issue for Bayesian and ML methods but it can still be a potential problem for genetic distance-based methods i.e. GBLUP and RKHS., presumably because of increased uninformative markers in calculating the genetic kinships. For the nonlinear phenotypes, high dimensionality still doesn’t affect ML until we have sufficiently large main effects ( Figures 2A and 2B; Extended Data: Figure S2B, Figure S3B and Figure S4B *,* ^27^). Here, for the case when main effects were sampled from a Gaussian distribution, increasing polygenicity is analogous to having many small main effects; so, despite having epistatic effects, performance goes down for all methods. In the nutshell, this shows that the conclusions drawn in Section ‘ML methods perform well for simple traits’ and Section ‘ML methods outperform parametric methods for complex traits’ holds under high-dimensionality.

**Case study in wheat**

**Figure 3.**
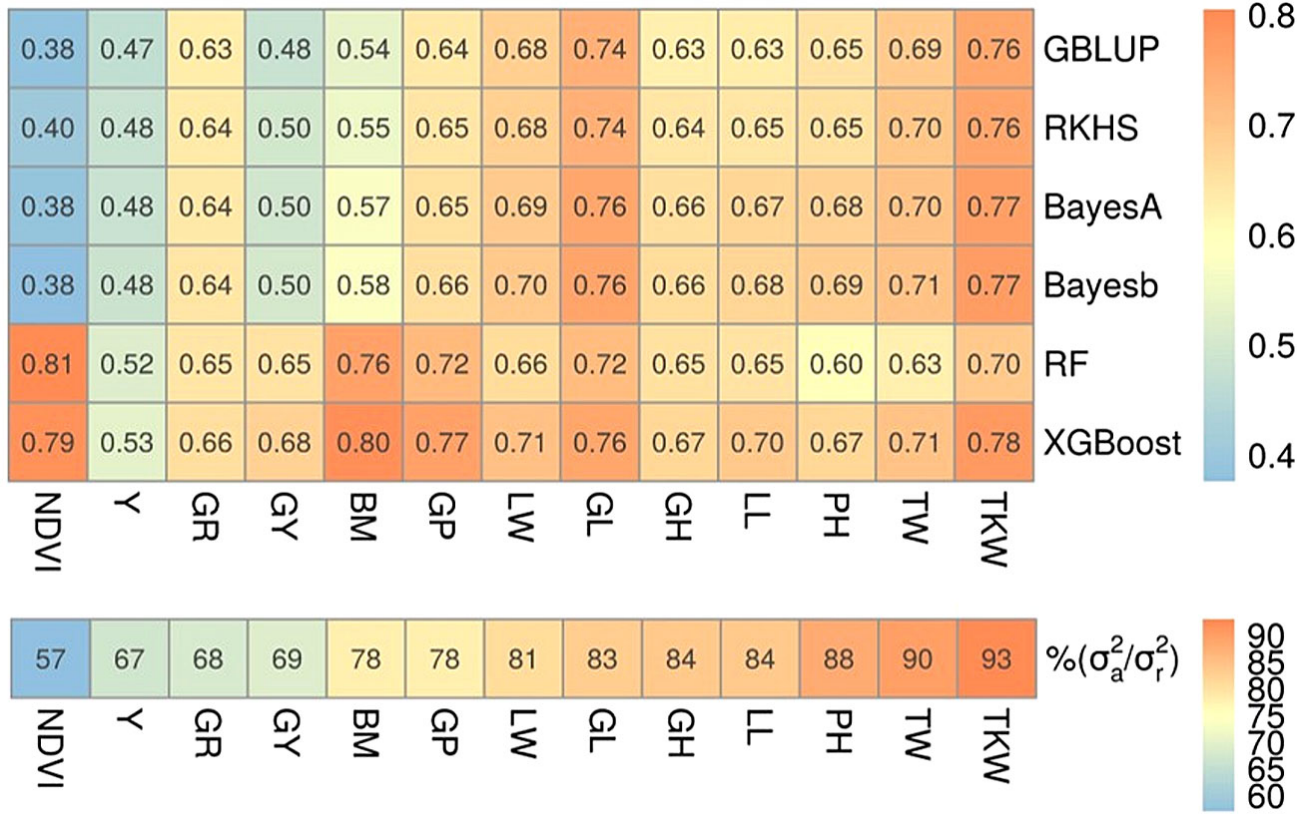
Prediction accuracies of wheat traits. Top: prediction accuracies for GP models on wheat traits, reported as the mean Pearson correlation coefficient ( *r*) of 5-fold cross-validation. Trait abbreviations are given in Table 2. Bottom: fraction of additive to residual genetic variance calculated using equation (9) for each trait. Traits were sorted in ascending order of additive variance fraction (left to right); therefore, the leftmost trait (NDVI) can be considered more complex than those to the right.

To see whether our simulation results hold on real traits, we used a dataset of 13 wheat traits ^24^ for a fairly large number of samples (10,375 lines) and 17,181 markers ( *c* ≈ 1.6). These markers have been selected by strict screening criteria, therefore, many of them could be informative. Insights in the genetic complexity for some of these traits were previously reported in Norman, Taylor ^24^ and Norman, Taylor. ^25^ For example, glaucousness was reported to be a simple trait, but grain yield to be more complex. ^25^ The results in Figure 3 clearly indicate that five-fold cross-validated prediction accuracies ( *r*) were higher for both ML methods when the fraction of additive variance was small (i.e. traits were fairly complex) and slightly lower or comparable to both Bayesian and GBLUP/RKHS regression methods otherwise. This is in line to what we observed in our simulations: for simple traits ( Figure 2A and B) ML performance was either comparable to Bayesian or slightly poorer, but for complex traits it was consistently better ( Figure 2C). For example, leaf width, glaucousness, growth habit, leaf loss, plant height, test weight and thousand kernel weight traits had greater than 80% of their genetic variance explained only by additive variance components and performance of ML relative to Bayesian methods and GBLUP/RKHS regression was either at par or lower than that. On the other hand, biomass, grain protein, grain yield, yellowness and in particular NDVI had smaller fractions of additive variance and, relative to the other methods, ML performed better. Hence, results on this experimental dataset match with the findings in our simulations that ML is best suited for the prediction of more complex traits and a potential candidate for simple traits as well.

### ML methods are sensitive to population structure

Population structure (PS) is a well-known confounding factor that results in decreased diversity in training populations ^25^ and unrealistic inflated parameter estimates, e.g. for (co) variances of random effects in LMMs ^51^ https://paperpile.com/c/ZyQHHy/ByqH. Parametric and nonparametric ML methods, based on their modelling assumptions and approaches, may be differently sensitive to PS. To assess the impact of population structure on ML methods, we used real genotype data with a known population structure and combined it with both simulated (STRUCT-simdata) and real phenotypes (STRUCT-realdata). Only linear phenotypes were simulated, with varying complexity and dimensionality scenarios, as described earlier in Section ‘Simulations’. The STRUCT-simdata contains all 1,307 *Arabidopsis* RegMap accessions. ^26^ To exclude the impact of multicollinearity among SNPs, only uncorrelated markers were retained after pruning with pairwise squared correlation coefficient ( *r* ^2^ < 0.1, see Section ‘Population structure analysis’), leaving 15,662 SNPs, but keeping the population structure intact (Extended Data, Figure S7 ^27^). This results in a ratio *c* = *p/n* of approximately 12 (15,662/1,307), a setting comparable to the simulation results presented in Figure 2A *.*

Correction for PS was carried out by including the top ten principal components corresponding to the largest eigenvalues as fixed effects into the mixed model equations or as additional features for ML methods. For the simulated phenotypes (Extended Data, Figure S6 ^27^), average pairwise difference of test accuracies before and after correcting for PS was slightly higher for ML methods (RF: 0.03 and XGBoost: 0.04) than for LMMs (GBLUP: 0.01, RKHS: 0.01, BayesA: 0.01 and BayesB: 0.00). Moreover, the correction resulted into relatively elevated accuracies for the scenarios with larger number of QTNs or low heritabilities. This illustrates that with smaller #QTNs and larger heritabilities ( *h* ^2^ = 0.7, #QTNs = 5), effect sizes per QTN were larger; therefore, confounding due to PS was less of a concern. With the decrease in effect sizes per QTN (increase in #QTNs and decrease in *h* ^2^), correction became more important for reliable predictions. From this, we can argue that confounding due to PS should be generally corrected for, but particularly for complex phenotypes having low heritability and large numbers of QTNs with small-medium effect sizes.

**Figure 4.**
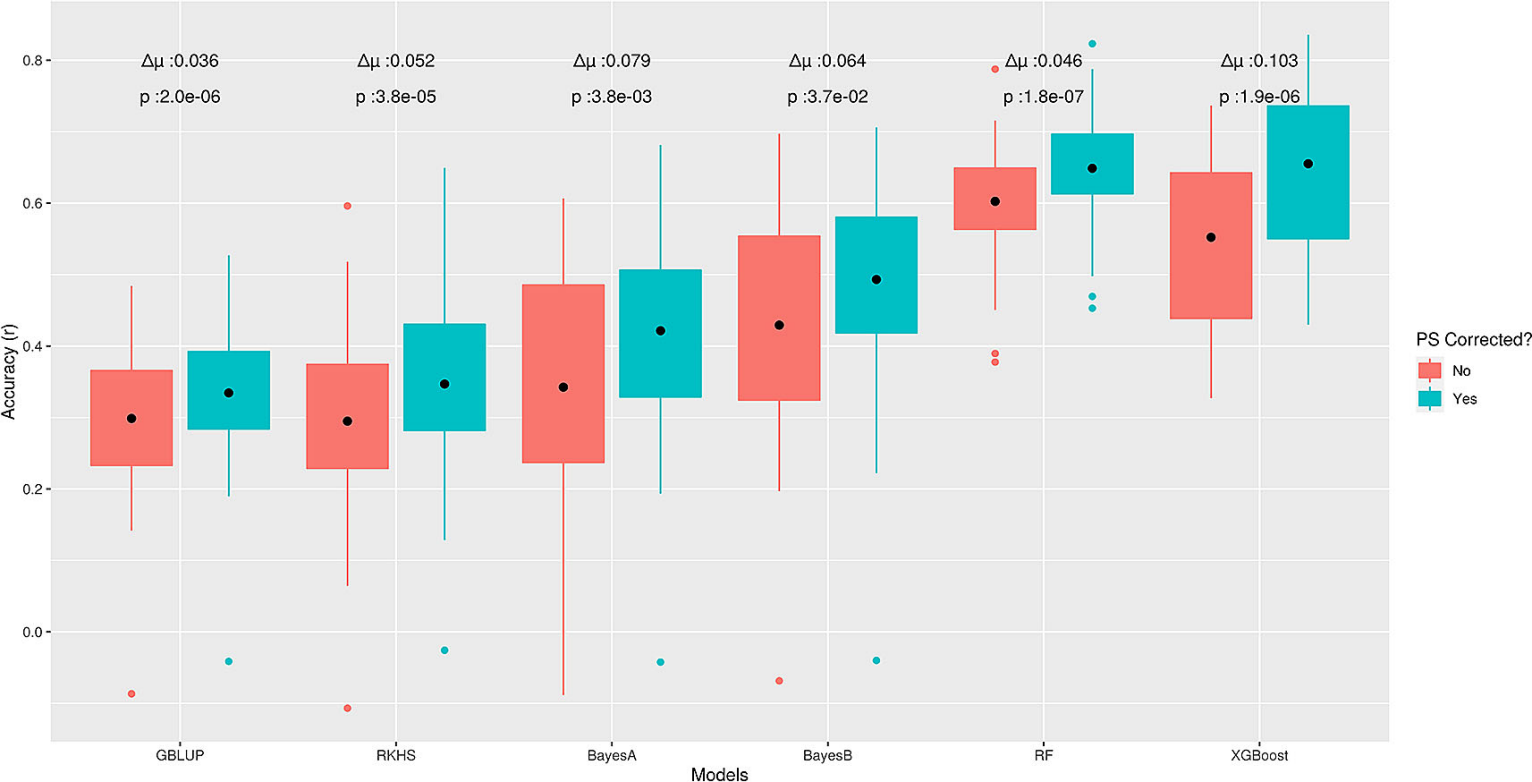
Effect of correction for population structure for the sodium accumulation trait in *Arabidopsis thaliana.* Boxplots present Pearson correlation coefficients ( *r*) found in *5*-fold cross-validation, on test data from ‘STRUCT-realdata’. Here ∆μ is the average difference between pairwise predictions before and after correction and for each model, the nonparametric Wilcoxon rank sum test was used to assess statistical significance.

To further explore this behaviour, we used real phenotypes of the sodium accumulation trait in *Arabidopsis thaliana* (STRUCT-realdata) using a subset of the same genotypes dataset. Here, we expected to have at least one large effect QTN for this trait, because *AtHKT1;1* locus, encoding a known sodium (Na ^+^) transporter, has been reported to be a major factor controlling natural variation in leaf Na ^+^ accumulation capacity. ^29^ Similar to the outcomes on ‘STRUCT-simdata’, correction for PS increased prediction accuracies of all methods on test data; whereas, GBLUP showed the lowest average difference (∆μ = 0.03) in performance before and after correction ( Figure 4). In contrast to ‘STRUCT-simdata’, XGBoost had the largest average difference (∆μ = 0.1) but for RF the difference was comparable to LMMs (∆μ = 0.05). From the above outcomes, we conclude that ML methods, like other GP methods, are sensitive to confounding due to PS and correcting for this can further improve performance for complex phenotypes. However, it is still unclear to which extent or for which GP problem characteristics different methods are more advantageous or more sensitive to PS.

### ML methods can tackle low SNP-QTN LD

The utility of GP in genomic selection is based on the assumption that there are ample markers within a densely genotyped set of markers which are in LD with the QTLs. ^1^ The actual QTNs are generally unknown, but SNPs in LD can be used to (partially) capture their effect, depending on the actual correlation and allele frequencies. Therefore, it is worthwhile to investigate the impact of SNP-QTN correlation levels on GP performance ^52^ https://paperpile.com/c/ZyQHHy/Q1iWE. We used two settings, one with real genotypes and simulated phenotypes (LD-simdata), a second with real genotypes and real traits (LD-soy).

**Figure 5.**
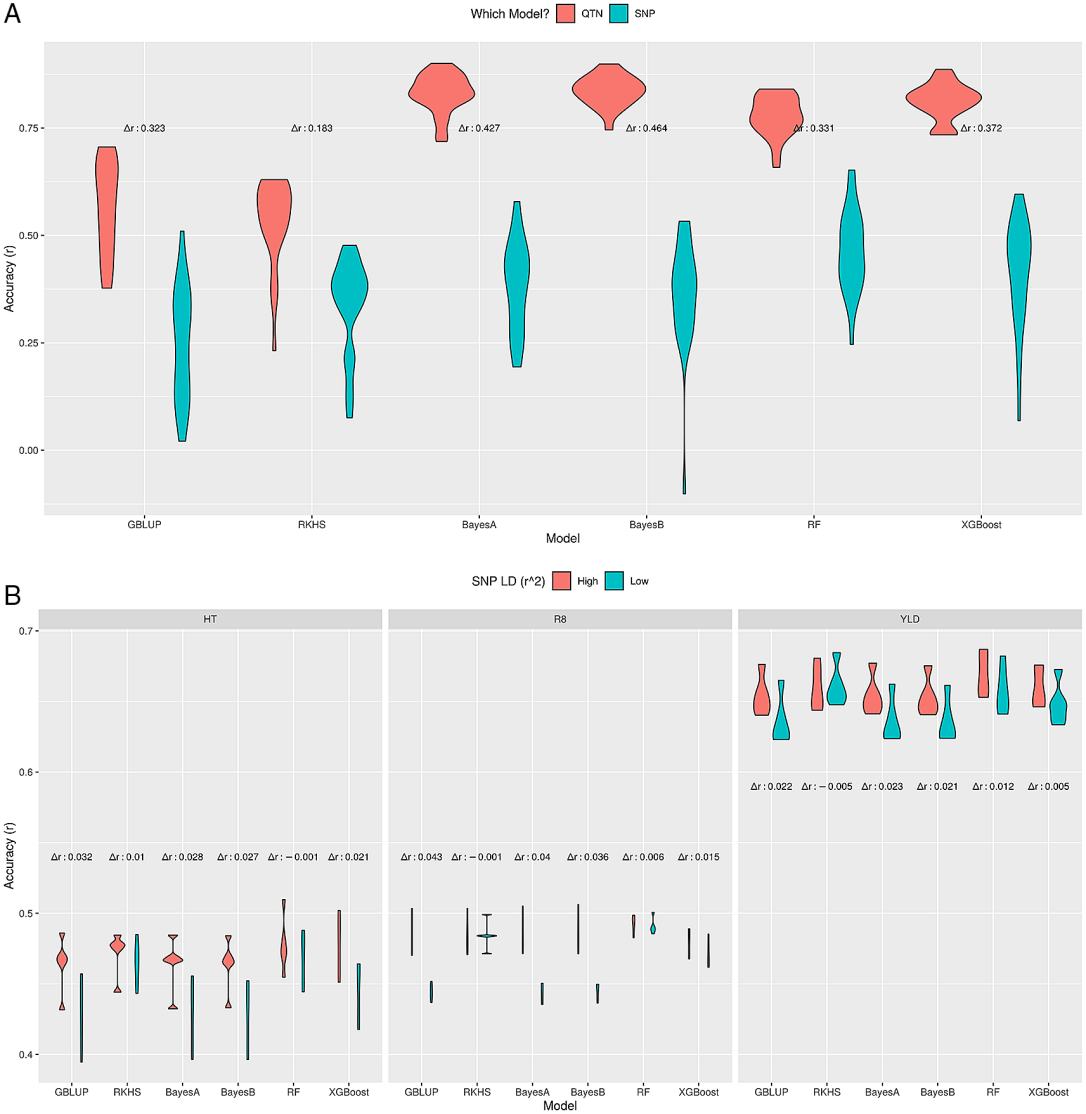
Effect of SNP-QTN LD on prediction accuracy. Prediction accuracy of different GP methods on simulated (A) and real soybean (B) datasets for high and low LD between SNPs and actual QTNs. The difference in median accuracies between these scenarios is indicated as Δ *r.* A) LD-sim data, low SNP-QTN LD ( *r* ^2^ ≤ 0.5). B) LD-soy data, low ( *r* ^2^ ≤ 0.5) SNP-QTN LD vs. all SNPs (high LD).

In simulations, GP model performance is evaluated based on the difference in prediction accuracies between a model trained on the actual QTNs and a model trained on SNPs in LD (QTN-linked SNPs). Our results show that when SNPs are highly correlated to QTNs (which is likely the case for densely genotyped markers set and *r* ^2^ > 0.9), all methods perform equally well and the SNP-based model predictions are very close to those of the actual QTN based models (Extended Data, Figure S8 ^27^). On the other hand, for low LD between SNPs and QTNs, there was in general a difference between median prediction accuracies (Δ *r*) of the QTN and SNP-based models ( Figure 5A). This difference varied between methods, from 0.18 for RKHS regression to 0.43–0.46 for the Bayesian methods, with GBLUP and ML methods between these (0.32–0.37). The relative robustness of particularly the Random Forest model in these circumstances compared to the Bayesian methods, in combination with its good performance in many simulations, supports its usefulness for GP.

As a real genotype and phenotype dataset, we used three Soybean traits, i.e. height, time to R8 developmental stage and yield (LD-soy). The complete set of markers (4,235 SNPs) had many correlated SNPs, such that only 261 were left with low LD ( *r* ^2^ ≤ 0.5). Here, in contrast to LD-simdata where we knew the QTNs in advance, we assumed that many SNPs could be linked to QTNs, because ~94% of all markers had *r* ^2^ > 0.5. So, we compared two models: one with all markers (the benchmark model), and one with low LD ( *r* ^2^ ≤ 0.5). A similar pattern was observed, as shown in Figure 5B, i.e. RKHS regression, RF and XGBoost were most robust against low SNP-QTN LD, with negligible differences between median accuracies, where GBLUP and the Bayes methods had higher differences.

In conclusion, GP methods that model SNP-QTN or SNP-SNP relation as a nonlinear function (RKHS, RF, XGBoost) were more stable under low SNP-QTN LD compared to other methods (GBLUP, BayesA, BayesB). Moreover, RF seems to couple good prediction performance with reliability under low SNP-QTN LD.

## Discussion

### There is room for ML in genomic prediction

Genomic prediction has long been the realm of parametric methods, but recently nonlinear supervised ML methods have become increasingly popular. Yet literature is unclear on the characteristics of GP problems that warrant application of ML methods. This study fills this gap and concludes that nonlinear tree-based ensemble ML methods, especially Random Forests, can outperform traditional methods for simple as well as complex polygenic traits where nonlinear allele effects are present. Moreover, ML methods are robust to high dimensionality, although further improvements, e.g. statistical or prior knowledge driven regularization, may improve performance. ML methods are particularly useful compared to the frequently used GBLUP and RKHS regression given their higher performance. While Bayesian methods often perform on par with ML models, this is mainly when there are large effect QTNs and/or linear phenotypes. Moreover, Bayesian methods are prone to overfitting in case of small sample sizes ( *p*/ *n* > 1), which is less of an issue with ML, especially with RF (Extended Data: Figures S9A and S9B ^27^).

### Tree-based ensemble ML methods are a reasonable choice for GP

A wide range of parametric, semi-parametric and nonparametric methods can be used for GP, but it is impractical to test all for a particular application. The choice for a suitable method strongly depends on the GP problem characteristics, described in Figure 1. While GP methodology can be compared using various model evaluation metrics (BIC, AIC, log likelihoods), we focused on their utility from a breeder’s perspective, so we compared only their prediction accuracies. We found that GP methods based on modelling the distance between genotypes using covariance structure(s), inferred from genomic markers (GBLUP and RKHS), were generally inferior to Bayesian and ML methods and less robust to high-dimensional problems likely because all of the *p* SNPs were used always to calculate the kinship matrices, whereas, either 5, 50, 100, *p*/2 or exactly *p* SNPs were chosen as QTNs. When *q* is fairly less than *p,* makes the kinship matrix too noisy due to the large number of markers that are unrelated to the phenotype but are used in the calculation of the GRM. Hence, we expect equal accuracies for increasing number of QTNs ( *q*), keeping the other factors ( *p, n* and *h* ^2^) fixed. Figure S5 (Extended Data ^27^) clearly illustrates that these methods indeed have constant prediction accuracies with increasing *q* values, while the accuracies of the other methods drop due to decreasing effect sizes. This further explains that their performance can be improved by removing unrelated markers from the GRM, for instance using biological knowledge about markers. ^31^^,^^53^

The parametric LMM equations can be solved using a Bayesian framework. Bayesian methods define prior SNP effects distributions to model different genetic architectures. Instead of a single distribution for all marker effects (e.g. BRR), it could be defined for each individual marker (e.g. BayesA). Mixture distributions have also been proposed (e.g. BayesC, BayesB). From the Bayesian alphabet, we used BayesA and BayesB as representatives because the first scenario, i.e. a single distribution for all markers, has been covered by GBLUP. Our results illustrate that these methods outperform GBLUP and RKHS regression when large effect QTNs are present, for both linear and nonlinear phenotypes. On the other hand, tree-based ensemble ML methods had either comparable performance to Bayesian methods (for simple traits) or superior performance (for complex traits). Capitalising on the results from Appendix-I (Extended data ^27^) that these ML methods had better performances than other ML methods (SVR and MLP), we can argue that these tree-based ML methods are a reasonable choice to conduct GP.

### Population structure analysis

Population structure can affect GP performance. Our results show that without correcting for population structure, test accuracies were lower than after correction for all methods. However, ML seems to be slightly more sensitive because the average difference between each pairwise test data accuracies was higher than other methods in the simulated data.

Confounding due to population structure can also be due to the frequently employed random cross-validation strategy for predictive modelling. ^25^ In random cross-validation, the reference population is randomly divided into subsets, one of which is iteratively selected for testing while the remaining subsets are used to train the model. While samples are all part of a test set once, under population structure some subpopulations may be over or under-represented in the training set. As a result, the model may get overfitted. A solution could be to use stratified sampling instead. On the other hand, parameter estimation may get misguided by within subgroup allele frequency differences rather than the overall true phenotype associated variance.

The impact of population structure can be dealt with in many ways. Conventionally, principal components of the SNP dosages or genomic relationship matrix are introduced as fixed effects in the mixed model equations. ^54^^–^^56^ Alternatively, phenotypes and genotypes can be adjusted by the axis of variations before predictive modelling. ^5^ Nevertheless, some residual structure often remains in the datasets, so it is important to check sensitivity of GP models to this confounding factor. Since ML methods (RF and XGBoost) do not employ any statistical prior and learn the association patterns from the data itself, they may be more sensitive to structure, as we found in our simulation results. But this is not clearly evident from the real phenotypes, so we cannot generalize this conclusion from our simulations.

### Effect of SNP-QTN linkage disequilibrium

Despite technological improvements, low density SNP panels are usually cost-effective for routine genomic selection. Increasing marker density does not necessarily increase prediction accuracy, since accuracy is not a linear function of SNP density only. ^57^^–^^59^ Instead, many GP problem characteristics ( Figure 1) jointly affect performance. However, using low density SNP panels can negatively affect prediction performance, since relevant SNPs in LD with the QTLs can either be completely missing or SNPs only in low LD may be present. As a result, allele frequencies between SNPs and QTNs can be quite different, resulting in incorrect predictions. ^52^ Despite this, low SNP density can still be sufficient for populations with larger LD blocks, e.g. F2 populations, where QTL detection power is highest and in this case, we shouldn’t expect much improvement by increasing marker density. But it becomes an important consideration when LD starts to decay and population relatedness decreases in the subsequent crosses of the breeding cycle. In this context, our study addresses the question of whether certain GP methods, especially ML, are more sensitive to low SNP-QTL LD. The results using both simulated and real traits indicate that SNP-QTL LD could also be an important determinant of suitable GP methodological choice and that ML is robust against low LD.

A weak SNP-QTL correlation implies that the SNP is a weak predictor of phenotype and there is an imperfect match between the genotypic distribution and the actual underlying genetic distribution of the phenotype. When using penalized regressions, this can result in different shrinkage for the SNP than that required by the actual QTN, thereby leading to a low genetic variance attribution to that SNP. Therefore, we may expect better prediction by nonparametric ML methods, as they may better learn weak genetic signals and are more robust to low SNP-QTL LD problems. On the other hand, the semiparametric RKHS regression method, which measures genetic similarity between individuals by a nonlinear Gaussian kernel of SNP markers, also performed better than GBLUP and Bayesian methods under low SNP-QTN LD. The reason could be that under low SNP-QTN LD, true pair-wise genetic covariance estimation would be less accurate due to losing many important markers and considering all of them equal contributors towards total genetic covariance. In case of RKHS regression, a Gaussian distribution defines a SNP’s probable contribution towards total genetic covariance, which becomes more realistic in this scenario because fewer important SNPs are left than in the high SNP-QTN LD case. The Bayesian methods (BayesA and BayesB) had the largest decrease in test performance under low SNP-QTN LD compared to high SNP-QTN LD. This could be due to the application of penalties on individual marker effects, which shrinks the weak SNP-QTN associations towards zero for each SNP.

### ML outperformed parametric methods for predicting complex wheat traits

Bread wheat breeding has huge impact on worldwide food security and socio-economic development. ^60^ Therefore, minor improvements in GP methodology leading to overall genetic gain can have high impact. In this study, we used a large (10,375 lines) Australian germplasm panel, genotyped with a high quality custom Axiom ^TM^ Affymetrix SNP array and phenotyped for multiple traits with varying complexity levels. ^24^ The authors showed that genomic selection was superior to marker-assisted selection (MAS) by employing GBLUP with two random genetic components (referred to as full-model in their text). Our results clearly indicate that ML can perform well for complex bread wheat traits, e.g. grain yield, yellows, greenness, biomass and NDVI. All of these traits except grain yield can be measured using high-throughput automated phenotyping. ^61^ This is an interesting finding since, with the rapid advances in low cost high-throughput phenotyping systems, attention is shifting towards measuring component traits, e.g. vegetative indices, rather than final yields. ML methods can predict these traits more accurately, as evident from our analysis.

## Conclusions and outlook

Based on simulated and real data, we conclude that tree-based ensemble ML methods can be useful for GP for both simple and complex traits. Moreover, these methods can work for both low- and high-density genotyped populations and can be a first choice for practical plant breeding. However, proper correction for population structure should be applied to obtain stable accuracies. Between bagged (Random Forests) or boosted (XGBoost) decision tree ensemble methods, Random Forests seem to be a good first choice for GP given their generalization performance and ability to work with high dimensional genotype data. It would be interesting to investigate to what extent these ML methods can benefit from statistical or prior knowledge-based regularization techniques.

## Data availability

### Underlying data

All datasets analysed during the current study are already published and publicly available ^22^^,^^23^ and references to their authors or repositories have been mentioned in the text.

### Extended data

Figshare: Extended data for ‘Genomic prediction in plants: opportunities for ensemble machine learning based approaches’.

This project contains the following extended data:

‐ Supplementary Figures: http://www.doi.org/10.6084/m9.figshare.19919002 ^25^
  • Figure S1. Assessment of phenotypic class (linear or nonlinear).
  • Figure S2. Comparison of test data prediction performance using simulated phenotypes with equal effects QTNs.
  • Figure S3. Comparison of test data prediction performance using simulated phenotypes with unequal effects QTNs.
  • Figure S4. Comparison of test data prediction performance using simulated phenotypes with QTN effects sampled from Gaussian distribution.
  • Figure S5. Effect of increasing number of QTNs to the total number of SNPs ratio on prediction performances using simulated phenotypes linear phenotypes.
  • Figure S6. Effect of population structure correction on GP model accuracies.
  • Figure S7. Principal Component Analysis (PCA) of *Arabidopsis thaliana* RegMap 1,307 accessions using uncorrelated set of markers.
  • Figure S8. Effect of high SNP-QTN LD ( *r* ^2^>0.9) on prediction accuracy.
  • Figure S9. Comparison of training data prediction performances using simulated phenotypes with one large effect QTN.
  • Figure S10. Comparison of prediction performances of parametric, semi-parametric and ML methods using simulated phenotypes without a large effect QTN for nonlinear phenotypes.
‐ Supplementary Tables: http://www.doi.org/10.6084/m9.figshare.19918729 ^46^
  • Table S1. Simple Traits
  • Table S2. Complex Traits
  • Appendix 1: Selection of machine learning (ML) candidates for genomic prediction.
http://www.doi.org/10.6084/m9.figshare.19919023 ^43^

## Software availability

Source code available from: https://git.wur.nl/faroo002/pub2

**Archived at** the **time of publication:** **https://doi.org/10.5281/zenodo.6734259** . ^23^

License: GPL version 3
