# Supplementary material for: Genomic prediction in plants: opportunities for ensemble machine learning based approaches
Source: F1000Res. 2023 Jan 10;11:802. Originally published 2022 Jul 18. [Version 2] doi: 10.12688/f1000research.122437.2 (PMC10080209; doi:10.12688/f1000research.122437.2)
Supplement: Peer review history

# Peer review: Reviewer response for version 2

**DOI:** 10.5256/f1000research.142631.r167303

**Published:** 2023-04-06

**Authors:** Yongkang Kim

**Affiliations:** 1 Institute for Behavioral Genetics, University of Colorado Boulder, Boulder, Colorado, USA

The paper descries comparison study of performances between various types of machine learning models and statistical models to find the best model for genomic prediction using SNP data. To compare the prediction performance, simulation was performed with several different ratio of causal and non-causal variants, the number of samples and variants, heritability, effect size distribution, existence of population stratification, LD structure, and different genomic assumptions. In the simulation study, ML methods generally performed better than statistical models. 

 The paper seems written well and the structure looks good. However, I think that it needs some more improvement, and there are some my suggestions below.

 First, the paper seems little bit long, so it would be better to reduce the length of paper by putting some introduction of methods to supplement. Also, I think that discussion section can be reduced by merging first, second, and fifth section of discussion.

 Second, in many prediction model studies, the marker selection step before constructing main model is essential to improve the prediction ability of statistical model. I think that if you perform SNP screening first, parametric models would perform better than current results. See Shigemizu *et al* ^1^.

 Third, it would be great to know how much time will take for each model development with various settings. I think that RKHS regression takes much longer times than the other methods so that it would be one of the great reason why ML need to be used for efficient prediction modeling.

 Fourth, in population structure analysis, I think that you need to calculate principal components with only training dataset and projects the PCs for testing dataset with those results. 

 I don't think authors need to do all of above suggestions to index the paper, but those things should be described in the paper.

Is the work clearly and accurately presented and does it cite the current literature?

Yes

If applicable, is the statistical analysis and its interpretation appropriate?

Yes

Are all the source data underlying the results available to ensure full reproducibility?

Yes

Is the study design appropriate and is the work technically sound?

Yes

Are the conclusions drawn adequately supported by the results?

Yes

Are sufficient details of methods and analysis provided to allow replication by others?

Yes

Reviewer Expertise:

I am working in statistical genetics area with statistical modeling and machine learning.

I confirm that I have read this submission and believe that I have an appropriate level of expertise to confirm that it is of an acceptable scientific standard, however I have significant reservations, as outlined above.

## References

[1] : The construction of risk prediction models using GWAS data and its application to a type 2 diabetes prospective cohort. *PLoS One* .2014;9(3) : 10.1371/journal.pone.0092549 e92549 10.1371/journal.pone.0092549 24651836PMC3961382

# Peer review: Reviewer response for version 1

**DOI:** 10.5256/f1000research.134423.r153250

**Published:** 2022-10-20

**Authors:** Miguel Pérez-Enciso

**Affiliations:** 1 Department of Animal Production, Universitat Autònoma de Barcelona, Barcelona, Spain

Authors present an ample study on ML and linear methods for GP. Their conclusion is very clear (RF and XGBoost end to outperform linear methods); However, they overinterpret, - RF and related methods are better when a few variables are of large influence, as multiple studies have shown. It is true that ensemble methods tend to be a safe choice (see eg Azodi *et al., *2019 ^1^). As many studies have shown, the best method is trait and scenario dependent. Therefore, I suggest to include a small dose of caution in the conclusions. The paper is perhaps overlong.

 I am not sure a clear rationale on choosing between ML and LMM is lacking, but you do not solve it.

 The term 'non linear phenotypes' is misleading, if it refers to the degree of non additivity, I am not sure we can tell so easily between linear and non linear phenotypes.

 A clear link between simulated and real phenotypes is often missing?

 The simulation of allele frequencies is completely unrealistic; not detailed on how effects are sampled.

 Table 1: you use up to 60k QTLs?

 Table 2, provide also n and p.

 GBLUP can also include dominance and epistasis (work by Vitezica, Varona, *et al., *2018 ^2^).

 What are the 'I ^th^' components in equation 6?

 Equation 9, not clear how genetic residual is calculated nor its meaning? The estimated non additive variance?

 A negative relation by the way of additive and non additive part cannot be generalized.

 Out of all scenarios simulated, the most realistic is panel F (Fig 2), which shows similar behavior across methods, as usually observed.

 Fig 3: weird some very big differences (e.g., NDVI phenotype). 

 Some review refs

- Azodi *et al. *(2019) ^1^
- Reinoso-Peláez *et al.* (2022) ^3^

Is the work clearly and accurately presented and does it cite the current literature?

Partly

If applicable, is the statistical analysis and its interpretation appropriate?

Yes

Are all the source data underlying the results available to ensure full reproducibility?

Yes

Is the study design appropriate and is the work technically sound?

Partly

Are the conclusions drawn adequately supported by the results?

Partly

Are sufficient details of methods and analysis provided to allow replication by others?

Yes

Reviewer Expertise:

Quantitative Genetics, Machine Learning

I confirm that I have read this submission and believe that I have an appropriate level of expertise to confirm that it is of an acceptable scientific standard, however I have significant reservations, as outlined above.

## References

[1] : Benchmarking Parametric and Machine Learning Models for Genomic Prediction of Complex Traits. *G3 GenesGenomesGenetics* .2019;9(11) : 10.1534/g3.119.400498 3691-3702 10.1534/g3.119.400498 PMC682912231533955

[2] : Non-additive Effects in Genomic Selection. *Front Genet* .2018;9: 10.3389/fgene.2018.00078 78 10.3389/fgene.2018.00078 29559995PMC5845743

[3] : Genome-Enabled Prediction Methods Based on Machine Learning. *Methods Mol Biol* .2022;2467: 10.1007/978-1-0716-2205-6_7 189-218 10.1007/978-1-0716-2205-6_7 35451777

# Response

**Published:** 2022-12-26

**Authors:** MUHAMMAD FAROOQ

**Affiliations:** Wageningen University and Research Centre, The Netherlands

We thank the reviewer for constructive criticism and providing a valuable input to the article that helped us to improve it further. We have tried to address the comments one by one and made some changes to the text accordingly.

**Reviewer) I suggest to include a small dose of caution in the conclusions. I am not sure a clear rationale on choosing between ML and LMM is lacking, but you do not solve it.**

**Authors) **We agree that the choice of a particular method is scenario dependent. We also concur that despite simulating many scenarios, we do not find a definitive rationale for choosing between ML and LMM. Instead, our study could provide some general guidelines that can be helpful when considering this issue. We have revised the conclusion section to clarify this and modified the abstract to avoid over-interpretation.

**Reviewer) The term 'non-linear phenotypes' is misleading, if it refers to the degree of non-additivity, I am not sure we can tell so easily between linear and non-linear phenotypes.**

**Authors) **Thank you for pointing this out. Although we did define non-linear phenotypes as those exhibiting epistatic interactions, we agree the use of this term can be misleading. We have therefore replaced all uses of linear and non-linear phenotypes by additive and epistatic, respectively.

**Reviewer) The simulation of allele frequencies is completely unrealistic; not detailed on how effects are sampled.**

**Authors) **We fixed MAF=0.4 and decided not to incorporate the impact of allele frequencies in our analysis because MAF of QTLs can impact heritability estimation and ultimately prediction accuracies (Yoshinobu Uemoto *et al.*, 2015). This allowed us to observe equal and reasonably enough statistical power for each SNP during allele effects estimation. To clarify for the readers, we have also mentioned this in the text now (lines 105-109).

 The effect sizes were sampled from a Gaussian distribution ~ *N*(0, √ *h* ^2^), to simulate three different cases. For the first case, one effect *β* was sampled and then all *q* QTNs were allocated *β _i_ = β* / *q*, i.e. all QTNs had equal effects. This allowed us to evaluate a trait complexity scenario where effect sizes could decrease with increasing numbers of QTNs. In the second case, we sampled two effect sizes (a large effect for a single QTN, a smaller effect for all other QTNs) from the effect size distribution. For the third case, all effects were randomly sampled from the Gaussian distribution. The Methods section now contains a more elaborate description of our approach on lines 130-141.

**Reviewer) Table 1: you use up to 60k QTLs?**

**Authors) **Yes; for the case in which we had 60k SNPs, one possible scenario was to use 60k QTNs. This was simulated to illustrate the infinitesimal modelling scenario.

**Reviewer) Table 2, provide also n and p.**

**Authors) **Thank you for the suggestion. Table 2 has been updated.

**Reviewer) GBLUP can also include dominance and epistasis (work by Vitezica, Varona, *et al., *2018** **).**

**Authors) **Indeed it can, and there are many other variations. We believed a full comparison of these methods to be out of the scope of this manuscript. However we did use RKHS as a general class of BLUPs with a three-kernel averaging scheme (De los Campos *et al.*, 2010) using bandwidth values b={0.2,0.5,0.8}.

**Reviewer) What are the 'I ^th^' components in equation 6?**

**Authors) **The RKHS method contains three random genetic effects. The i ^th^ component is the i ^th ^random genetic effect, with a genomic relationship matrix determined by Gaussian kernels corresponding to i = first, second or third value of bandwidth from {0.2,0.5,0.8}. We clarified this on line 294.

**Reviewer) A clear link between simulated and real phenotypes is often missing?**

**Authors) **We agree that a direct link between simulated and real traits is difficult to establish because simulations simplify things to a large extent. A better link could perhaps be established if the same population was utilised for simulated phenotypes. However, the purpose of our simulations was to give a general idea of where RF/XGB could be a potential choice, not for a specific population. We observed these methods work well for the case with a few QTNs with large effect or when the phenotype contains interaction effects. When the trait is highly polygenic and additive, such that the effect sizes distribution resembles a Gaussian ( *Figure 2*), similar performance can be expected for RF/XGB and LMMs.

**Reviewer) Equation 9, not clear how genetic residual is calculated nor its meaning? The estimated non additive variance? A negative relation by the way of additive and non-additive part cannot be generalized.**

**Authors) **We agree that this was not clear and have changed equation (9) to accommodate the additive and epistatic random genetic components explicitly. Thus, the additive component is governed by the additive covariance matrix, whereas the epistatic component has a covariance matrix accommodating a fraction of the SNP interactions, as proposed by the E-GBLUP methodology (Yong Jiang and Jochen C Reif 2015). With both of these random components, we were able to extract the proportion of additive (σ ^2^ *_a_*) and epistatic (σ ^2^ *_e_*) variances for a trait and therefore, the σ ^2^ *_a_*/σ ^2^ *_e_*, should in principle, be higher when epistatic variance is low and vice versa. We have mentioned this in the text for clarification on lines 349-355. This negative relation was also found in our simulated data ( *Figure S1*). Accordingly, for the real wheat traits, we observed a negative relation between σ ^2^ *_a_*/σ ^2^ *_e_ *and the maximum difference between LMMs and RF/XGB accuracies. There was a clear separation between the traits, as plotted in *Figure R1*, with a lower additive:epistatic variance ratio (e.g. GY, GP, NDVI, BM etc) and those with a higher ratio (e.g. TW, TKW, GL, PH, LW).

 We agree that this negative relation cannot be generalized, but given our results testing the modified equation (9) on multiple phenotypes of the same population, we believe it to be an extrapolation for the same population. In the above *Figure R1*, we can see that for traits that are predominantly additive and polygenic, LMMs and RF/XGB perform quite similar (as for the cases in Figure 2-panel F); but for traits with a predominant epistatic component, for example, grain yield and biomass, RF/XGB outperform LMM.

**Reviewer) Out of all scenarios simulated, the most realistic is panel F (Fig 2), which shows similar behavior across methods, as usually observed.**

**Authors) **We agree to some extent that F is more realistic for many complex traits, but the scenarios in panels B and C can also be observed, e.g. for oligogenic traits. An example is the sodium accumulation trait ( *Figure 4*), where ML performed well due to the large effect QTN. The panels with equal effects were meant (as a theoretical exercise) to illustrate the impact of many small effect QTNs and small narrow-sense heritabilities. We have clarified this point further in the text on lines 658-666.

**Reviewer) Fig 3: weird some very big differences (e.g., NDVI phenotype).**

**Authors) **The large difference in prediction accuracy for grain yield and biomass could be attributed to their larger epistatic variance compared to the other traits. For the NDVI trait, phenotypic variance and narrow-sense heritability were low in this dataset, so this should be specific to this dataset only. We have explicitly mentioned this issue on lines 773-775 in the text.

# Peer review: Reviewer response for version 1

**DOI:** 10.5256/f1000research.134423.r145166

**Published:** 2022-09-28

**Authors:** Muhammad Tehseen Azhar

**Affiliations:** 1 Department of Plant Breeding and Genetics, University of Agriculture Faisalabad, Faisalabad, Pakistan

All the components are written according to international standards and latest information is cited. ​​​​​

 The statistical designs are used according to the demand of hypothesis and nature of data for the interpretation of the results. The protocols are elaborated in this manuscript so that one can repeat these experiments for more interpretations. The needed statistical analyses are conducted for the interpretation of the data.

 Methods-Data-Simulations: Explain the reason why you selected 500 for the sample size. What was the rationale behind choosing fixed MAF=0.4 and not other than that?

 In Figure 5B, why does yield have more accuracies than height and R8; whereas, yield is usually considered as a relatively more complex trait than others.

 The data is submitted in the relevant repository and authors would be available to answer any query from the researchers.

 The conclusion is to the point based on results and will be guideline for later studies. The Ref#55. Patterson N, Price AL, Reich D: Population Structure and Eigen analysis. PLoS Genet. 2006; 2(12): e190. should be removed because Ref#54 is its follow up study.

Is the work clearly and accurately presented and does it cite the current literature?

Yes

If applicable, is the statistical analysis and its interpretation appropriate?

Partly

Are all the source data underlying the results available to ensure full reproducibility?

Partly

Is the study design appropriate and is the work technically sound?

Yes

Are the conclusions drawn adequately supported by the results?

Yes

Are sufficient details of methods and analysis provided to allow replication by others?

Partly

Reviewer Expertise:

Plant Breeding and Biotechnology, Genetics

I confirm that I have read this submission and believe that I have an appropriate level of expertise to confirm that it is of an acceptable scientific standard.

# Response

**Published:** 2022-12-26

**Authors:** MUHAMMAD FAROOQ

**Affiliations:** Wageningen University and Research Centre, The Netherlands

We thank all reviewer for providing a valuable input to the article that helped us to improve it further. We have tried to address their comments one by one and made some changes to the text accordingly.

**Methods-Data-Simulations: Explain the reason why you selected 500 for the sample size. What was the rationale behind choosing fixed MAF=0.4 and no other than that?**

 We chose 500 samples since a reference population of this size is common for many genomic selection applications in plant breeding, as mentioned on lines 112-113. For example, Nonoy *et al*., 2022 used 457 soybean breeding lines, Sandhu *et al.*, 2021 used 650 spring wheat lines etc. We fixed MAF=0.4 for all SNPs, and decided not to incorporate the impact of allele frequencies because the MAF of QTLs can impact SNP heritability estimation and ultimately prediction accuracies (Yoshinobu Uemoto *et al.,* 2015). With this setting, we obtained enough statistical power for each SNP during allele effects estimation (line 105-109).

**In Figure 5B, why does yield have more accuracies than height and R8; whereas, yield is usually considered as a relatively more complex trait than others.**

 We used the ~4.2k SNPs extracted by Azodi *et al*., 2019 from the complete SoyNAM genotype dataset after rigorous feature selection. The motivation was to choose a real, low-dimensional dataset with highly correlated SNPs to understand the impact of SNP-QTL LD only, while benchmark accuracies were also available in that study. Note that in general the accuracy of a genomic prediction model depends on whether and how many of the SNPs causal for a trait are included in the model. It might be that the feature selection performed by Azodi *et al. *had a stronger impact on height and R8 compared to yield. In any case, our results do match with those of Azodi *et al.,* 2019 (Figure 5A), who found lower accuracies for predicting height and R8 than for yield, given this selected subset. We thank the reviewer for pointing this out, and now explicitly mention this in the text on lines 636-638 and 645-646.

**The Ref#55. Patterson N, Price AL, Reich D: Population Structure and Eigen analysis. PLoS Genet. 2006; 2(12): e190. should be removed because Ref#54 is its follow up study.**

 Thank you for pointing it out. This reference has now been removed.
